# Comparison of Motor Inhibition in Variants of the Instructed-Delay Choice Reaction Time Task

**DOI:** 10.1371/journal.pone.0161964

**Published:** 2016-08-31

**Authors:** Caroline Quoilin, Julien Lambert, Benvenuto Jacob, Pierre-Alexandre Klein, Julie Duque

**Affiliations:** Institute of Neuroscience, Université catholique de Louvain, Brussels, Belgium; University of Ottawa, CANADA

## Abstract

Using instructed-delay choice reaction time (RT) paradigms, many previous studies have shown that the motor system is transiently inhibited during response preparation: motor-evoked potentials (MEPs) elicited by transcranial magnetic stimulation (TMS) over the primary motor cortex are typically suppressed during the delay period. This effect has been observed in both selected and non-selected effectors, although MEP changes in selected effectors have been more inconsistent across task versions. Here, we compared changes in MEP amplitudes in three different variants of an instructed-delay choice RT task. All variants required participants to choose between left and right index finger movements but the responses were either provided “in the air” (Variant 1), on a regular keyboard (Variant 2), or on a response device designed to control from premature responses (Variant 3). The task variants also differed according to the visual layout (more concrete in Variant 3) and depending on whether participants received a feedback of their performance (absent in Variant 1). Behavior was globally comparable between the three variants of the task although the propensity to respond prematurely was highest in Variant 2 and lowest in Variant 3. MEPs elicited in a non-selected hand were similarly suppressed in the three variants of the task. However, significant differences emerged when considering MEPs elicited in the selected hand: these MEPs were suppressed in Variants 1 and 3 whereas they were often facilitated in Variant 2, especially in the right dominant hand. In conclusion, MEPs elicited in selected muscles seem to be more sensitive to small variations to the task design than those recorded in non-selected effectors, probably because they reflect a complex combination of inhibitory and facilitatory influences on the motor output system. Finally, the use of a standard keyboard seems to be particularly inappropriate because it encourages participants to respond promptly with no means to control for premature responses, probably increasing the relative amount of facilitatory influences at the time motor inhibition is probed.

## Introduction

Appropriate human behavior entails the ability to choose actions in a goal-directed manner. This human competence is thought to rely on a fine interplay between excitatory and inhibitory processes that shape motor activity during action preparation [1]. The influence of these processes on the motor output system has been investigated by measuring the amplitude of motor evoked potentials (MEPs) elicited by single-pulse transcranial magnetic stimulation (TMS) applied over the primary motor cortex (M1) during choice reaction time (RT) tasks.

Studies focusing on motor inhibitory processes have often used choice RT tasks in which an instructed-delay period requires participants to select a response based on a preparatory cue (usually a movement of the left or the right hand) and then to withhold it until the onset of an imperative signal [2–6]. When TMS probes are applied at the end of the delay period in such tasks, MEPs are markedly attenuated with respect to baseline. Interestingly, this effect can be observed both when MEPs are elicited in a muscle that is required (selected effector) or not required (non-selected effector) for the forthcoming response. Converging lines of evidence suggest that these effects result from the operation of at least two different motor inhibitory mechanisms [2–5, 7], although alternative interpretations have been suggested in other works [8, 9]. A first mechanism is thought to suppress MEPs in selected effectors. Such process may be particularly critical during instructed-delay periods given the requirement to postpone the initiation of selected responses until the onset of the imperative signal. Besides, the MEP suppression observed in non-selected effectors is thought to be caused by a second inhibitory process assisting response selection. The latter would prevent inappropriate movement representations from becoming too active and thus from being erroneously selected. This second mechanism seems to exert a global inhibitory effect on motor activity. Hence, it is likely to also contribute to the MEP suppression observed in selected effectors. In agreement with this framework, MEP suppression is often (although not always) greater in a muscle selected for the forthcoming response compared with a non-selected effector [4, 10–12]. Notably, whereas the generic inhibitory form has been related to cortical processes regulated by the lateral prefrontal cortex [3], the specific inhibitory process is thought to operate at the spinal level and to rely on the premotor cortex [2, 13].

So far, most instructed-delay RT tasks have required participants to provide their response “in the air” by performing a finger movement in the absence of any key-press. In such tasks, performance is typically evaluated offline based on electromyography (EMG) traces and participants are not provided with any online feedback of their performance. Recently, we have asked the participants to deliver their response on a keyboard and have given them a feedback of their performance (accuracy and speed) at the end of each trial [12]. These changes did not impact on MEPs elicited in non-selected effectors; the latter were nicely suppressed, consistent with all previous reports [12]. In contrast, the MEPs elicited in selected effectors showed very little suppression in this context; they were even facilitated when elicited in the right dominant hand. Hence it seems that the way participants have to provide their response, and/or the presence of a feedback, impacts on the strength of the MEP suppression observed in selected effectors during the instructed-delay of choice RT tasks.

The goal of the present experiment was to compare the pattern and strength of MEP changes during the instructed-delay of three variants of a choice RT task. All variants required participants to choose between left and right index finger abduction movements but the responses were either provided “in the air” (Variant 1), on a keyboard (Variant 2), or on a response device designed to control from premature responses (Variant 3). Besides, a visual feedback was provided in Variants 2 and 3 (absent in Variant 1) and the visual layout was different (i.e. more concrete) in Variant 3 compared to the two other versions. We assessed the impact of these task features on MEPs elicited in a task-relevant (index finger abductor) and a task-irrelevant (fifth digit abductor) muscle.

## Materials and Methods

### Participants

A total of 12 right-handed volunteers (9 women; mean age = 21.67 ± 0.57 years old) participated in the experiment. Handedness was determined via a condensed version of the Edinburgh Handedness Inventory [14]. None of the participants suffered from any neurological disorder or had a history of psychiatric illness, drug or alcohol abuse; none either was undergoing any drug treatment that could influence performance or neural activity. Participants were naive to the purpose of the study and were financially compensated. They all gave written informed consent at the beginning of the study, following the protocol approved by the Ethics Committee of the Université catholique de Louvain (UCL), in compliance with the principles of the Declaration of Helsinki.

### Instructed-delay choice RT task

Participants sat in front of a computer screen, positioned about 60 cm in front of them. They performed three different variants of an instructed-delay choice RT task, which was implemented with Matlab 7.5 (The Mathworks, Natick, Massachusetts, USA) and the Cogent 2000 toolbox (Functional Imaging Laboratory, Laboratory of Neurobiology and Institute of Cognitive Neuroscience at the Wellcome Department of Imaging Neuroscience, London, UK). The task consisted in a virtual “soccer game” in which participants had to shoot a ball into a goal as fast as possible, using the left or the right hand. The required response was indicated by the position of a preparatory cue, with participants being instructed to respond with an abduction of the left index finger when the cue was displayed on the left side of the screen and with the right index finger when it was presented on the right. Importantly, the cue allowed them to prepare their movement but they were required to wait for the onset of an imperative signal before they could initiate it.

#### Visual layouts and response devices used in the three variants of the task

**Fig 1:**
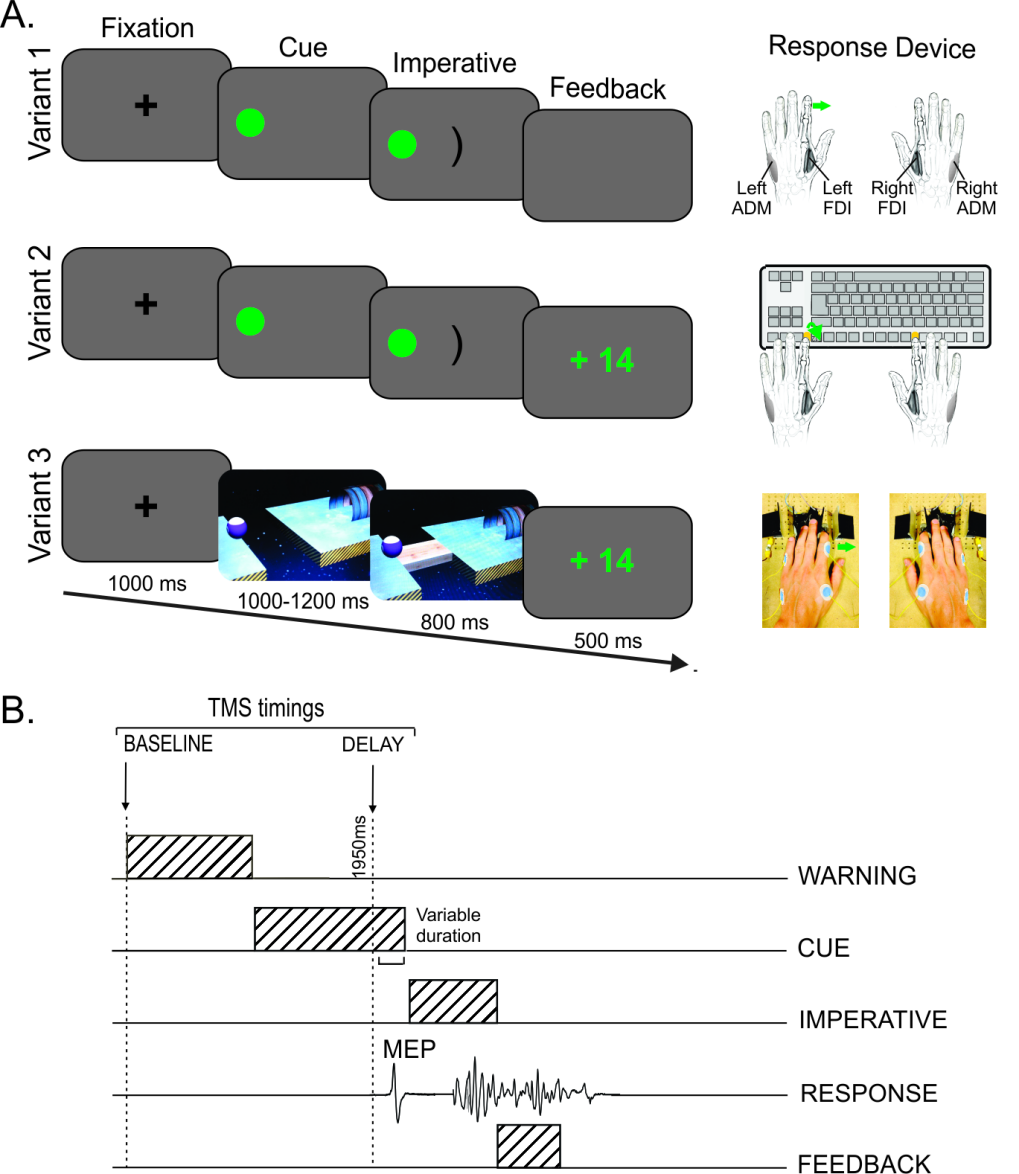
Experimental procedure. (A) Sequence of events and response devices in the three variants of the instructed-delay choice reaction time (RT) task. In all variants, participants were required to perform an abduction of the left or the right index finger in order to shoot a ball into a goal. The required response was indicated by the position of a preparatory cue that appeared on the left or the right side of the computer screen after a fixation cross (i.e. a left response in this example). Importantly, participants had to withhold their response until the onset of an imperative signal. This signal appeared after a random delay period of 1000 to 1200 ms and remained on the screen for 800 ms (Variant 1) or until a finger response was detected (Variants 2 and 3). Finally, a feedback was displayed for 500 ms. Please note the absence of feedback in Variant 1 (blank screen instead). The three variants of the task were implemented with different visual layouts and response devices. In Variant 1, the preparatory cue consisted in a filled green circle and the imperative signal was a central bracket oriented toward the circle. Participants were instructed to perform their movement “in the air”. The same visual display was used in Variant 2, but participants had to respond on a standard keyboard and received a feedback of their performance after each trial. Variant 3 involved concrete objects displayed with a 3D perspective: the preparatory cue consisted in a ball and a goal placed on two platforms separated by a gap, and the imperative signal was a bridge linking these two platforms. Participants had to perform their movement on a response device designed to control for the occurrence of premature responses. (B) TMS timings. One single TMS pulse was delivered in each trial at one of two possible timings: either at the onset of the fixation cross (at TMS_BASELINE_) or during the delay period (at TMS_DELAY_), 950 ms after the preparatory cue onset (thus falling 50–250 ms before the imperative signal). FDI = first dorsal interosseous. ADM = abductor digiti minimi. MEP = motor evoked potential.

The three variants of the instructed-delay choice RT task are depicted in Fig 1A.

Variant 1 has been used in many previous studies on motor inhibition [4, 9, 14]. In this variant of the task, the preparatory cue consisted in a filled green circle (the ball) and the imperative signal was a central bracket oriented toward the circle (the goal). No device was used to record the responses: participants had both hands resting on a pillow, palms down, and were instructed to perform their abduction movement “in the air”. In order to evaluate the responses, this setup required subsequent analyses of the EMG traces of the left and right first dorsal interosseous (FDI) muscles (agonist of index finger abduction) for each single trial.

Variant 2 of the task was identical to Variant 1 except that here, a standard keyboard was used to record the finger responses and a feedback of the performance was provided [12]. Participants placed their hands palms down on a keyboard turned upside down so that they could press on the required buttons with the left or right index fingers (keys “F12” and “F5”, respectively). Between each response, participants were asked to place their index fingers on two small rubber pads, which were positioned on the external side of the two target buttons. Hence, each key press required participants to perform a brisk abduction and flexion movement of the left or right index finger. The key-press information was used online to provide the participants with a feedback of their performance (speed and accuracy) at the end of each trial (see below).

Both the visual layout and the response device were changed for Variant 3. One important goal here was to make the task look more concrete and to better control for premature responses. For this reason, we used actual objects, all displayed with a 3D perspective. The preparatory cue was composed of a ball and a goal placed on two different platforms separated by a gap; the imperative signal consisted in a bridge linking these two platforms, allowing the ball to roll into the goal. The response device was developed in our laboratory and was designed to detect any horizontal movement of the index fingers. It was composed of two pairs of metal edges fixed on wooden supports (one for each hand) and the response required participants to move one index finger from the outer to the inner metal edge. As the metal parts are mobile, they were placed according to the morphology of each participant. The contact between the finger and the metal part was continuously monitored using a Makey Makey printed circuit board with an ATMega32u4 microcontroller running the Arduino Leonardo firmware, based on the principle of high resistance switching between two electrical contacts. To enhance connectivity, a thimble was placed on both index fingers. This device has two main advantages compared to a standard keyboard. First, it measures separately the RT (by considering the time at which the finger leaves the outer metal edge, so-called “departure time”) and the movement time (by considering the difference between the time at which the finger reached the inner metal edge, so-called “arrival time” and the departure time). Departure and arrival times were recorded by the microcontroller and sent to a computer through a serial interface. A second advantage is that it provides a means to control for (and penalize) the occurrence of premature responses. That is, the device permanently checks the initial index finger position (the finger has to be in contact with the outer metal edge) and can detect any contact release before the onset of the imperative signal: when this happens, the ball falls in the gap, aborting the trial. With such a system, participants are really forced to put the break on their responses. Obviously, regular keyboards do not allow exerting such a control on the participant behavior. Nonetheless, this point is critical in the current context as any anticipation will reduce our ability to detect inhibitory influences on MEPs elicited close to the imperative signal onset, especially in selected effectors. Finally, in this variant of the task, participants were provided with a feedback of their performance after each trial, similar to Variant 2 (see below).

Importantly, the participants were in a relaxed position in all three variants of the task, with both arms resting in a semi-flexed position, palms down. In addition, the use of a response device in Variants 2 and 3 helped to maintain the participants’ hands in a stable position during the entire experiment, preventing any changes in muscle length that could influence the MEP measurements. This aspect of the task was obviously less well controlled in Variant 1.

### Experimental procedure

The sequence and timing of events were similar in all variants of the task (see Fig 1). Each trial began with a small black fixation cross displayed at the center of the computer screen for 1000 ms (grey background). This signal was followed by the onset of the preparatory cue. After a random period of 1000 to 1200 ms, the imperative signal appeared and remained on the screen for 800 ms (Variant 1) or until a finger response was detected (Variants 2 and 3). To prevent participants from anticipating, the three variants of the task comprised a few catch trials (5.56% of trials) in which the preparatory cue was not followed by an imperative signal. Finally, in Variants 2 and 3, a feedback was presented in the middle of the screen for 500 ms. Following a correct response, the feedback consisted in a positive score depicted in green which was inversely proportional to the trial RT (*k*/√*RT* with *k* = 250 and a maximum score of 25). By contrast, participants were presented with a fixed negative score (-10) depicted in red when they responded with the incorrect finger, or when they performed their response too fast (before the imperative signal onset) or in the absence of imperative signal (catch trials). In addition, they were penalized when they responded too slowly (more than 800 ms after the imperative signal onset, fixed score = -5). In Variant 1, the feedback screen was replaced by a 500 ms blank screen. Finally, trials were separated by an interval of 2300 ms (blank screen).

The experiment began with a short practice period in order to familiarize the participants with the choice RT requirements in each variant of the task. In the main phase of the experiment, participants performed six blocks of 72 trials. Each variant was tested in two successive blocks; the order of these block sets was counterbalanced between participants. In addition, for each pair, TMS was applied over the left M1 (eliciting MEPs in the right hand) in one block and over the right M1 (eliciting MEPs in the left hand) in the other one. Again, the order of the TMS application was counterbalanced between participants. Each block consisted of an equal proportion of left and right hand trials (i.e. 36 trials per hand condition, 2 of which were catch trials). Blocks lasted approximatively 6–7 minutes, and participants were given a short break after every pair of blocks (i.e. between each variant of the task).

One TMS pulse was applied in each trial, with two possible timings. To establish a baseline measure of CS excitability, some trials involved TMS at the onset of the fixation cross (TMS_BASELINE_; 24 MEPs/block). In the remaining 48 trials, TMS was delivered during the delay period (TMS_DELAY_), 950 ms after the onset of the preparatory cue (i.e. randomly delivered 50 to 250 ms before the imperative signal; overall mean across all conditions and subjects = 149.1 ms, SD = 58.1 ms), when participants were withholding a left (24 MEPs/block) or a right (24 MEPs/block) hand response. At this timing, a MEP suppression occurring in the hand cued for the forthcoming response (i.e. selected condition, MEP_LEFT_ or MEP_RIGHT_ preceding a left or a right hand response, respectively) is thought to reflect a combination of both generic and specific inhibitory mechanisms, while a MEP suppression in the non-cued hand (i.e. non-selected condition, MEP_LEFT_ or MEP_RIGHT_ preceding a right or a left hand response, respectively) is thought to result only from the generic inhibitory form. Hence, this procedure allowed us to evaluate the suppression of MEP_LEFT_ and MEP_RIGHT_ in selected and non-selected conditions for each of the three variants of the task. Importantly, because the TMS pulse was applied at a fixed time with respect to the onset of the preparatory cue, MEPs were measured at a comparable stage of action preparation in all trials, even if the duration of the delay period was random (1000 to 1200 ms).

### TMS protocol

TMS pulses were generated with a figure-of-eight coil (wing external diameter, 70 mm) connected to a Magstim 200 magnetic stimulator (Magstim, Whitland, Dyfed, UK). The coil was placed tangentially on the scalp over M1_LEFT_ or M1_RIGHT_ with the handle pointing backward and laterally at a 45° angle away from the midline, approximately perpendicular to the central sulcus. For each M1, the optimal coil position for eliciting MEPs in the contralateral FDI was identified and marked on a head cap placed on the participant’s scalp to provide a reference mark throughout the experiment [15]. The resting motor threshold (rMT) was determined as the minimal TMS intensity required to evoke MEPs of about 50 μV peak-to-peak in the relaxed FDI muscle in 5 out of 10 consecutive trials. Across participants, the rMTs corresponded to 40.9 ± 1.5% and 42.5 ± 1.8% of the maximum stimulator output for M1_LEFT_ and M1_RIGHT_, respectively. For each hand, the intensity of TMS throughout the experiment was always set at 120% of the individual rMT.

### EMG recording

EMG activity was recorded in the left and right FDI and ADM (abductor digiti minimi) muscles from surface electrodes (Ambu Blue Sensor NF-50-K, Neuroline, Medicotest, Oelstykke, Denmark) placed over the belly of each muscle and the corresponding metacarpophalangeal joint. EMG data were collected for 3200 ms on each trial, starting 200 ms before the TMS pulse. The raw EMG signals were amplified (gain, 1K), online filtered (high-pass cut-off frequency of 3 Hz) and digitized at 2000 Hz for off-line analysis. The EMG signals from the FDIs and the ADMs were used to measure the peak-to-peak amplitude of MEPs, with ADM MEPs allowing to investigate changes in CS excitability occurring in an irrelevant muscle of the selected or the non-selected hand. Besides, the EMG signals from the FDIs were also used to determine the RTs, defined as the time interval between the appearance of the imperative signal and the onset of the movement. The latter was obtained automatically using an algorithm detecting a predefined change in the root mean square of the EMG activity. Trials with background EMG activity larger than 100 μV in the 200 ms window preceding the TMS pulse were excluded from the analysis. This was done to prevent contamination of the MEP measurements by significant fluctuations in background EMG [16–18]. Trials in which participants had provided the wrong response were also removed from the MEP data set. After trimming the data for errors, background EMG activity and outliers, a mean of 21.3 ± 2.37, 21.2 ± 3.42 and 20.2 ± 3.84 trials remained to assess MEPs in the FDIs (in each condition and across the subjects) for Variant 1, 2 and 3, respectively. Regarding the ADMs, a mean of 20.9 ± 3.52, 19.7 ± 4.66 and 20.8 ± 3.84 trials remained in the corresponding conditions. As such, 6.53 and 7.28% of the trials were excluded for the analyses performed on the FDI and ADM MEPs, respectively.

### Statistical Analyses

#### Behavior

Behavior was compared in the three variants of the task by using three main dependent variables: the RTs (based on the EMG signal, see above), the number of trials within which participants responded prematurely (anticipation errors) and the number of trials in which they provided a response in the absence of imperative signal (catch errors). The EMG RT data were analyzed using a four-way analysis of variance for repeated measures (RM ANOVA), with VARIANT (1, 2, 3), RESPONDING-HAND (Left, Right), TMS-TMING (Baseline, Delay), and MEP-CONDITION (whether MEPs were elicited in the responding hand or not: Hand_MEP_ and Hand_NO-MEP_, respectively) as within-subject factors. To analyze the errors (anticipation and catch), we used nonparametric statistics (Friedman tests) with VARIANT (1, 2, 3) as the within-subject factor. Indeed, the few number of catch trials (4/block), and the lack of homoscedasticity between each sub-condition for the anticipation errors required the use of a nonparametric test. Moreover, to increase the number of observations, left and right hand trials were pooled together for this analysis.

#### MEP measurements

In order to assess CS excitability during the task, similar analyses were performed on the FDI and ADM MEPs. First, we compared MEPs at TMS_BASELINE_ between the three variants of the task. The raw amplitude of these MEPs (mV) was analyzed using a two-way RM ANOVA, with VARIANT (1, 2, 3) and MEP-SIDE (Left, Right) as within-subject factors. Then, we considered MEPs at TMS_DELAY_; these MEPs were always expressed in percentage of those elicited at TMS_BASELINE_ in the same block type. These data were first analyzed with a three-way RM ANOVA, with VARIANT (1, 2, 3), MEP-SIDE (Left, Right) and CONDITION (Selected, Non-selected) as within-subject factors. Finally, in order to assess the significance of motor inhibitory effects in each sub-condition, one-sample t-tests comparing the normalized MEPs to a constant value of 100 (i.e. the baseline) were performed.

When appropriate, effect sizes were provided. Depending on the analyzes, we have either used Cohen’s d (t-tests), partial eta-square (ANOVAs), or a r statistic based on the z provided by post-hoc analysis using Wilcoxon signed rank tests conducted with a Bonferroni correction (nonparametric statistics). ANOVAs were followed by Newman-Keuls post-hoc comparisons. All of the data are expressed as mean ± SE. Analyses were carried out using Statistica 7 (StatStoft, Cracow, Poland).

## Results

### Behavior

Behavior was examined by considering the EMG RTs as well as the anticipation and catch errors. Two participants had to be excluded from the RT analysis because technical problems precluded us from recording enough trials in some of the experimental conditions (i.e. <12 trials in at least one condition); hence a total of ten participants were considered for this analysis.

**Fig 2:**
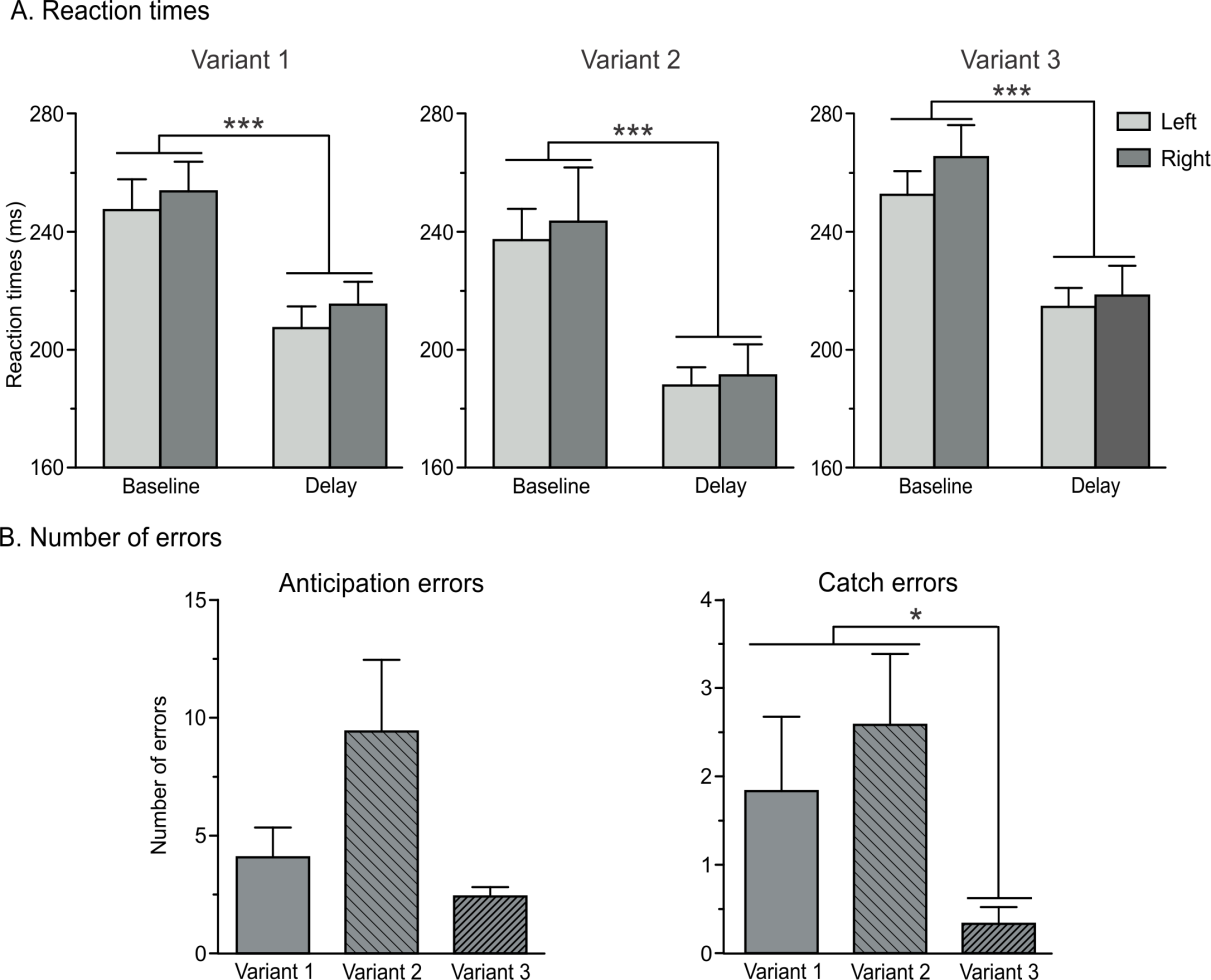
Behavioral performance. (A) Reaction times (ms) for responses performed with the left (light grey bars) and the right (dark grey bars) hands across the three variants of the task in trials at TMS_BASELINE_ and TMS_DELAY_. (B-C) Number of errors across the three variants of the task. Anticipation errors (B) refer to the number of trials in which participants responded before the onset of the imperative signal, whereas catch errors (C) correspond to trials in which a response was provided in the absence of the imperative signal. Data from left and right responses are pooled together. Note the tendency for errors to be more frequent in Variant 2, particularly when compared to Variant 3 (main effect of the factor VARIANT, p = 0.12 and p < 0.01 for anticipation and catch errors, respectively). *p<0.05 and ***p<0.001: significantly different.

Overall, the mean RTs were comparable in Variant 1 (231 ± 8 ms, *n* = 10), Variant 2 (215 ± 10 ms) and Variant 3 (238 ± 7 ms) of the task. RTs tended to be faster in Variant 2 but this effect was not significant (VARIANT F_2,18_ = 3.28, p = 0.06, η_p_² = 0.27; see Fig 2A). As evident on the figure, RTs were shorter with TMS_DELAY_ than with TMS_BASELINE_. As such, the factor TMS-TIMING was significant (F_1,9_ = 41.86; p < 0.001; η_p_² = 0.82). This result is consistent with many previous reports showing that a TMS pulse applied close to the imperative signal can speed up the release of a motor response [4, 8, 11]. This effect occurred regardless of whether a MEP occurred in the responding or in the non-responding hand (i.e. TMS-TIMING X MEP-CONDITION interaction; F_1,9_ = 0.67; p = 0.43; η_p_² = 0.07), and did not depend on the task variant or on the hand (left or right) used to provide the response (all F < 1.86 and p > 0.18).

The average number of anticipation errors equaled 4.08 (SE = 1.26, *n* = 12), 9.42 (SE = 3.04), and 2.42 (SE = 0.40) in Variants 1, 2 and 3, respectively. As shown on Fig 2B, participants tended to anticipate more in Variant 2 (when they had to provide their response by pressing a key on a keyboard), but this effect was not significant (VARIANT $X_{r}^{2}$ = 4.20; p = 0.12). In contrast, catch errors were found to differ between the three variants of the task (VARIANT $X_{r}^{2}$ = 13.19; p < 0.01) with catch errors being more frequent in Variants 1 and 2 than in Variant 3 (3D layout with home-made response device, both p < 0.05 and r > 0.46; Fig 2C).

Taken all together, the RT and error data suggest that the behavior was globally comparable between the three variants of the task although the propensity to respond prematurely was highest in Variant 2 and lowest in Variant 3.

### MEP measurements

**Fig 3:**
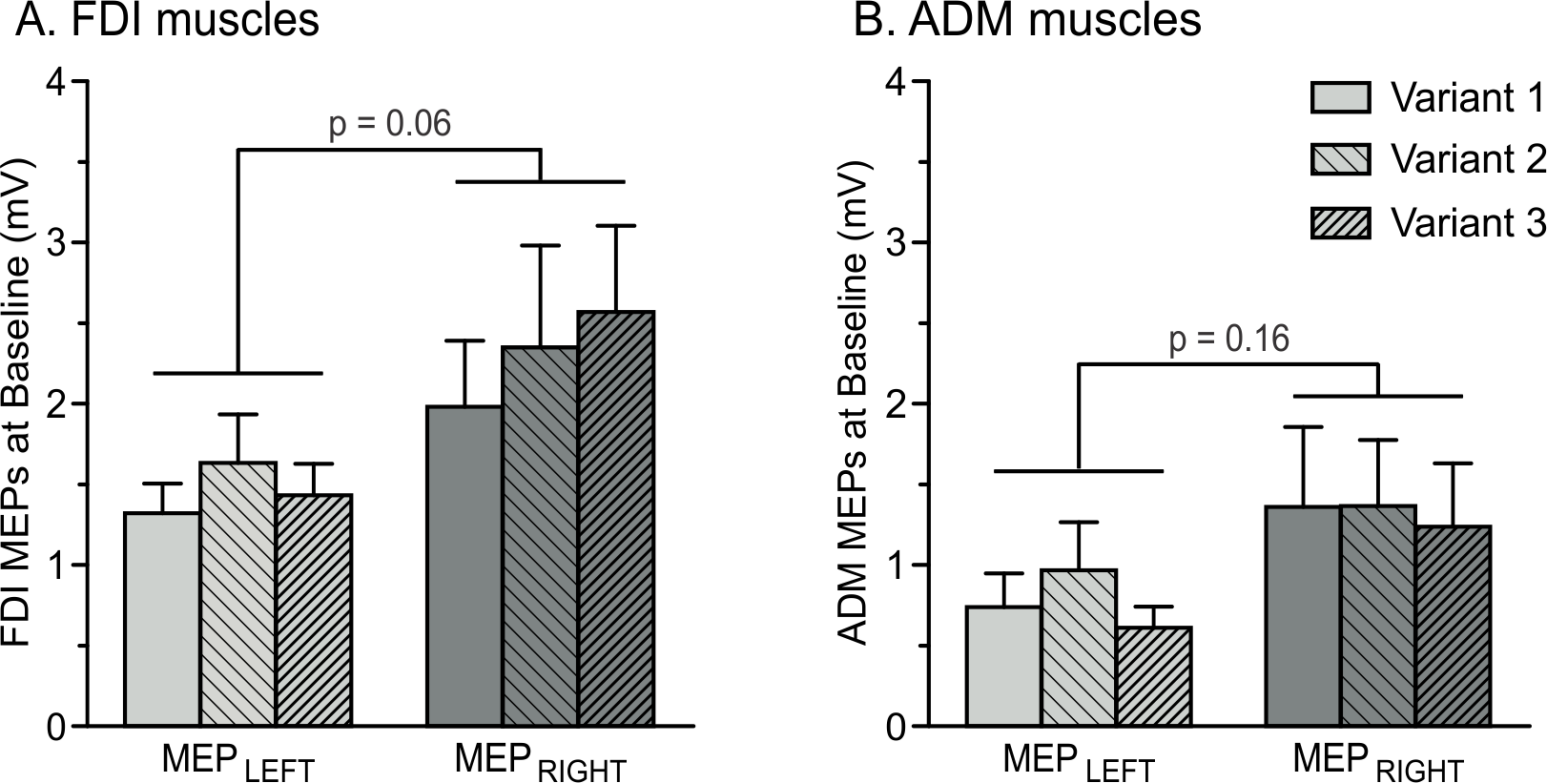
**Amplitude of MEPs (mV) recorded at TMS**_**BASELINE**_ **in the FDI (A) and ADM (B) muscles.** MEPs are shown for the left (MEP_LEFT_) and right (MEP_RIGHT_) hands across the three variants of the task. Note the tendency for MEPs to be larger in the right (dominant) compared to the left (non-dominant) hand, especially in the FDI muscles.

At TMS_BASELINE_, the mean amplitude of FDI MEPs was 1.46 mV (SE = 0.20, *n* = 12) and 2.30 mV (SE = 0.51) when elicited in the left and the right hands, respectively. Consistent with previous reports [12], MEPs tended to be larger in the right (dominant) hand than in the left hand (see Fig 3A), although this effect did not reach statistical significance (F_1,11_ = 3.67; p = 0.06; η_p_² = 0.25). Moreover, analyses revealed that FDI MEPs at TMS_BASELINE_ were comparable between the three variants of the task (main effect of the factor VARIANT; F_2,22_ = 0.96; p = 0.12; η_p_² = 0.18). Besides, ADM MEP amplitudes averaged 0.77 mV (SE = 0.20) and 1.32 mV (SE = 0.42) when elicited in the left and the right hands, respectively; they also seemed to be larger in the dominant than in the non-dominant hand. However, this effect did not reach significance (F_1,11_ = 2.26; p = 0.16; η_p_² = 0.17), neither did the factor VARIANT (F_2,22_ = 1.78; p = 0.19; η_p_² = 0.14; Fig 3B).

**Fig 4:**
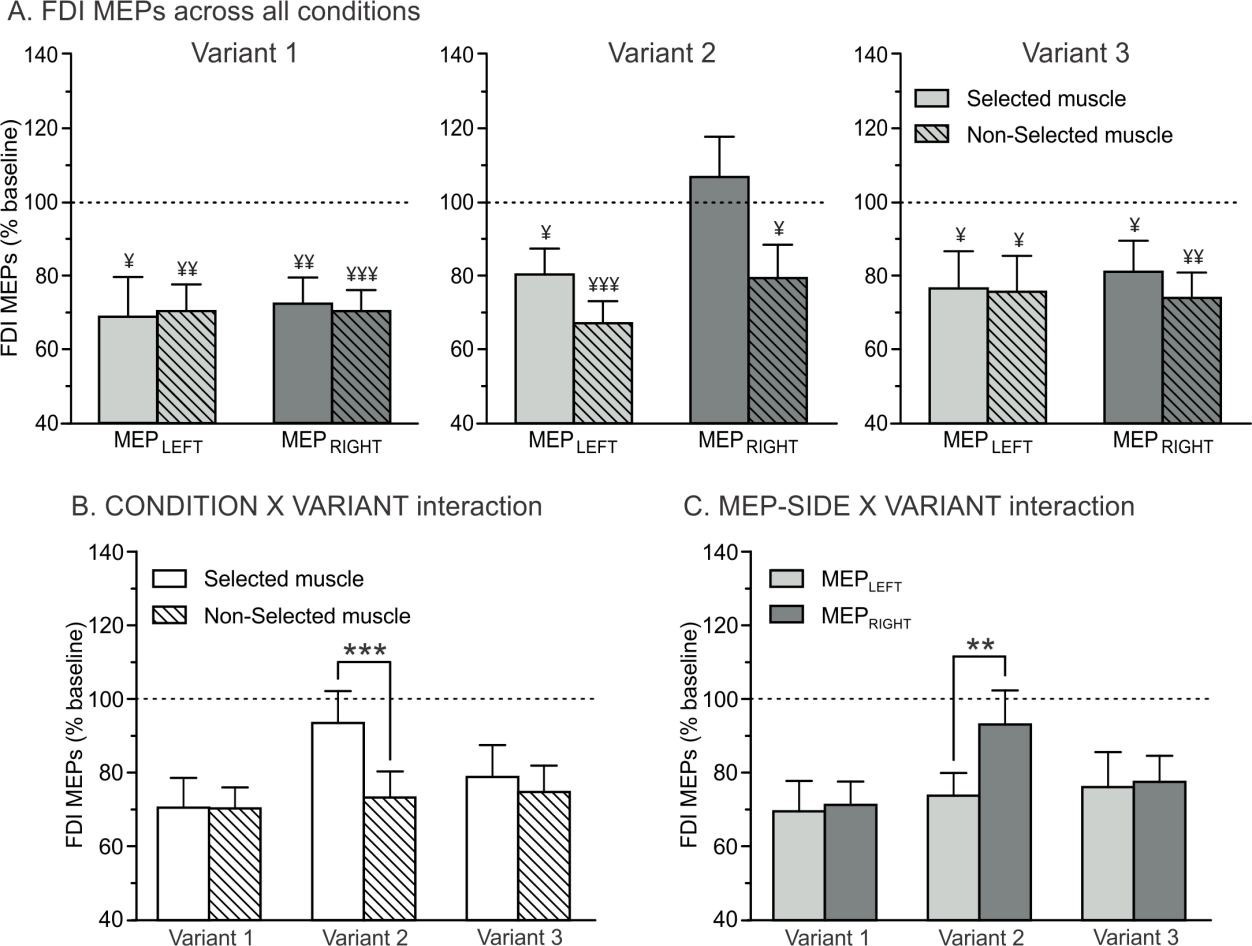
Amplitude of FDI MEPs recorded at TMS_DELAY_, expressed in percentage of MEPs elicited at TMS_BASELINE_. (A) MEPs are shown for the FDI in the left (MEP_LEFT_, light grey bars) and right (MEP_RIGHT_, dark grey bars) hands, when this hand was either selected (open bars, thought to reflect both generic and specific inhibition) or non-selected (dashed bars, thought to reflect generic inhibition only) for the forthcoming response in the three variants of the task. ¥ = significantly different from baseline (p<0.05, p<0.01 and p<0.001). (B) CONDITION x VARIANT interaction. FDI MEPs were larger in the selected than in the non-selected condition in Variant 2; such difference was not found in Variants 1 and 3. ***p<0.001: significantly different (C) MEP-SIDE x VARIANT interaction. FDI MEPs were larger in the right than in the left hand in Variant 2; such difference was not found in Variants 1 and 3. **p<0.01: significantly different.

Fig 4A shows the amplitude of MEPs elicited at TMS_DELAY_ (expressed in percentage of MEPs at TMS_BASELINE_) in the left and the right FDIs when this muscle was either selected or non-selected for the forthcoming response, for each of the three variants of the task. As evident on the figure, FDI MEPs at TMS_DELAY_ were suppressed in all conditions (significantly reduced with respect to baseline) except when they were elicited in a right hand that was selected for the forthcoming response in Variant 2 (selected condition t_11_ = 0.63; p = 0.54; d = 0.18 when compared to a constant value of 100; all other at least p < 0.05 and d > 0.64).

This different pattern of FDI MEP suppression in Variant 2 was further supported by a significant CONDITION x VARIANT interaction (F_2,22_ = 9.65; p < 0.001; η_p_² = 0.47; Fig 4B). As such, in Variants 1 and 3, MEPs were suppressed in a comparable way whether they were elicited in a selected or a non-selected condition (p = 0.95 and p = 0.25 when comparing conditions in Variants 1 and 3, respectively). By contrast, in Variant 2, MEPs were significantly smaller in the non-selected (73% of MEPs at TMS_BASELINE_) compared to the selected (94%) condition (p < 0.001). Note that the CONDITION x VARIANT x HAND interaction (F_2,22_ = 0.47; p = 0.63; η_p_² = 0.04) was not significant, suggesting that the attenuated MEP suppression in the selected condition of Variant 2 was present whether probed in the left (MEP_LEFT_) or right (MEP_RIGHT_) hand.

Our analyses also revealed a significant MEP-SIDE X VARIANT interaction (F_2,22_ = 3.57; p < 0.05; η_p_² = 0.24; Fig 4C). Again, this result was due to a different pattern of changes in Variant 2. As such, MEP suppression was greater when probed in the left compared to the right FDI (p < 0.01) in Variant 2, while this difference was not present in Variants 1 and 3 (all p>0.75). No further main effects or interactions were found to be significant.

**Fig 5:**
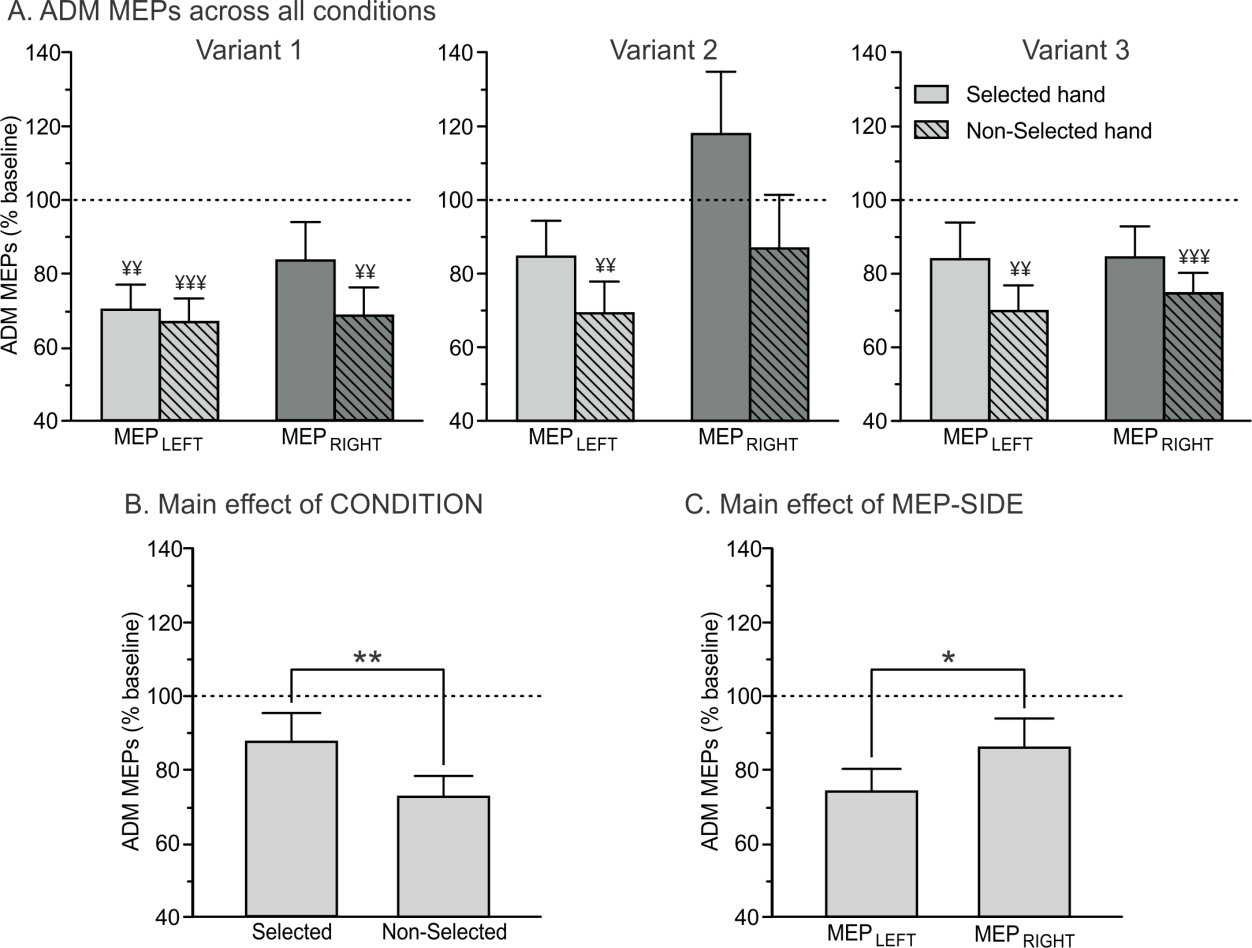
Amplitude of ADM MEPs recorded at TMS_DELAY_, expressed in percentage of MEPs elicited at TMS_BASELINE_. (A) MEPs are shown for the ADM in the left (MEP_LEFT_, light grey bars) and right (MEP_RIGHT_, dark grey bars) hand, when this hand was either selected (open bars, thought to reflect both generic and specific inhibition) or non-selected (dashed bars, thought to reflect generic inhibition only) for the forthcoming response in the three variants of the task. ¥ = significantly different from baseline (p<0.01 and p<0.001). Our results revealed significant main effects of the factors CONDITION (B) and MEP-SIDE (C). *p<0.05 and **p<0.01: significantly different.

As evident on Fig 5A, motor inhibition was also observed in the ADM, although this muscle was never recruited to perform the movements. However, the pattern of MEP suppression in this task-irrelevant muscle was slightly different from the one observed in the FDI. That is, we observed main effects of CONDITION (F_1,11_ = 10,47; p < 0.01; η_p_² = 0.49; Fig 5B) and MEP-SIDE (F_1,11_ = 4.81; p = 0.05; η_p_² = 0.30; Fig 5C), regardless of the variant of the task (CONDITION x VARIANT and MEP-SIDE x VARIANT interactions; all F < 3.17and p > 0.07): ADM MEPs were generally larger (i.e. less suppressed) in the selected than in the non-selected condition. Besides, they were globally larger in the right dominant hand than in the left non-dominant hand.

## Discussion

Most TMS experiments have reported motor inhibitory changes during the instructed-delay of choice RT tasks. This effect is very reliable and constant across studies when the MEPs are elicited in a non-selected effector. There have been more inconsistencies regarding the MEP changes occurring in a selected effector. Whereas some studies have reported a suppression of MEPs, others have observed a MEP facilitation, although the task characteristics were largely similar in all these works. Yet, some differences existed, including the use (or not) of response devices and the presentation of feedback screens; the latter features may have unexpectedly impacted on the task requirements and thus on MEP amplitudes. The main goal of the present study was to compare the pattern and strength of MEP changes during the instructed-delay of three variants of a choice RT task.

Variant 1 of the instructed-delay choice RT task has been routinely used in the past [2, 5, 10, 19]. It requires participants to perform left or right index finger abduction movements “in the air” according to the position of a circle presented on the left or the right side of the computer screen. Consistent with previous studies, we observed a strong suppression of MEPs during the delay period. However, contrary to most of the earlier works, the strength of this suppression was comparable whether MEPs were elicited in a muscle that was selected (e.g. MEP_LEFT_ preceding a left hand response) or non-selected (e.g. MEP_LEFT_ preceding a right hand response) for the forthcoming response. In fact, the selected condition seemed to be associated with less MEP suppression than usually reported in the past using a similar task version [2, 10, 11, 19]. Future experiments are clearly required to identify the causes of this discrepancy. The latter may include small variations in the duration of the delay period, the time of the TMS pulse and/or the predictability of the imperative signal onset. Notably, although the main task features of Variant 1 are comparable to those used in many previous studies, some subtle differences exist when considering the abovementioned parameters. Hence, small adjustments to these parameters may have impacted on MEP amplitudes, probably because they modify the relative contribution of inhibitory and facilitatory influences on MEP amplitudes, particularly when probed in a selected condition.

A comparable pattern of MEP suppression was observed in Variant 3 of the task. In this newly developed version of the instructed-delay RT task, MEPs were also reduced in the selected and non-selected conditions and, similar to the observation made in Variant 1, the magnitude of this suppression was equivalent in both conditions. Hence, our results indicate that this new version is appropriate to reproduce observations made with Variant 1. Yet, this variant of the task offers the considerable advantage of providing participants with concrete instructions and with observable consequences of their responses. In particular, an abduction of the correct index finger triggered the movement of a ball depicted on the screen; it made it roll on a bridge in order to reach a goal displayed on the other side, with the ball rolling faster following quicker responses. Furthermore, a positive feedback (inversely proportional to the RT) was presented after each successful trial. By contrast, a response initiated before the onset of the imperative signal led to the fall of the ball into the gap; in this case participants were provided with a negative score. Indeed, the response device was specifically designed to permanently control for any contact release before the appropriate time, the final purpose being to force participants to withhold their response until the imperative signal onset. Consistently, the amount of anticipation and catch errors was particularly low with this variant of the task. Thus, our results indicate that this newly developed version may be used to assess motor inhibition in delayed-response paradigms, provided that future studies determine the best combination of parameters to observe a larger MEP suppression in selected than in non-selected effectors, as reported in the past.

Finally, Variant 2 of the task used the same visual layout as in Variant 1 but participants provided their responses on a standard keyboard and received a feedback of their performance after each trial. Albeit less commonly used than Variant 1, this version of the instructed-delay RT task has been employed in the past, mostly because the keyboard offers an easy way to record the finger responses and to provide online feedback to the participants [12]. Consistent with the observations made in Variants 1 and 3, MEPs were strongly reduced when elicited in a non-selected condition. In contrast, the selected condition was associated with a particularly weak MEP suppression; it was much less pronounced than in the non-selected condition. In the past, a few studies have reported an increase in MEP amplitudes during delay periods [12, 20, 21]. Interestingly, all of them required participants to provide their response by pressing a button. Hence, it seems that the use of a keyboard can alter considerably the pattern of MEP suppression in a selected effector.

Such a finding draws our attention to the fact that MEPs represent global readouts of motor excitability and that their amplitude is influenced by multiple excitatory (or dis-inhibitory) and inhibitory processes acting at the same time [22, 23]. In particular, MEP changes probed in a muscle selected for the subsequent action are thought to reflect the contribution of two main processes. On the one hand, the cortical activity of the selected representation increases in order to prepare the system for the required forthcoming action, with the disinhibition of M1 potentially arising from a release of intracortical inhibition [24–26]. On the other hand, the spinal excitability associated with the selected response is inhibited, probably to prevent the engagement of the peripheral motor system during the delay period [2, 13], although alternative interpretations have been suggested[8, 9]. Thus, the attenuated MEP suppression observed in the selected condition of Variant 2 could be due either to the fact that withholding a keyboard response is associated with less inhibition than in the other versions, or to the fact that the degree of facilitation of the selected motor representations was higher at the time MEPs were probed in this specific condition. Based on the finding that participants tended to respond faster and made significantly more anticipation errors in this variant, we believe that the latter possibility is more likely. As such, participants might have been in a more advanced state of preparation at TMS_DELAY_, preventing us from observing the impact of inhibitory influences. Hence, it is likely that TMS occurred too close to the imperative signal in this variant of the task to identify inhibition of selected effectors. Future experiments are required to study precisely the evolution of inhibitory and facilitatory influences on MEP amplitudes during action preparation, probably by applying TMS at several time points during the instructed-delay of choice RT tasks [11].

Moreover, in this second variant of the task, the MEP suppression was weaker in the right dominant hand than in the left non-dominant hand. In fact, MEP suppression was even absent when the right hand was selected for the subsequent movement. These findings reveal some hemispheric asymmetry in CS excitability changes during preparation of a keyboard response. Again, a similar observation was made in our recent work using the exact same experimental setup [12], supporting the reliability of this effect. Yet, no hemispheric asymmetry was observed when participants had to provide their response “in the air” using Variant 1 of the task [19]. Hence, a hemispheric asymmetry was only disclosed in a task characterized by a lower degree of MEP suppression in the selected effector, possibly because the facilitatory influences were particularly strong when participants had to respond with their dominant hand [19]. Noteworthy, MEPs elicited at baseline also tended to be of higher amplitude in the right dominant hand relative to the left hand, despite the fact that a slightly lower stimulation intensity (lower rMT) was used to stimulate the dominant M1. This finding is in line with many previous reports [19, 27] and corroborates the view that handedness may be related to a more excitable motor cortex in the left hemisphere of right-handed individuals [19, 27, 28].

Finally, we also evaluated CS excitability changes occurring in a muscle that was not involved in the RT task. That is, although the TMS hotspot was defined based on the best location to induce a response in the FDI, we could also record MEPs in the ADM (abductor muscle of the fifth digit) when stimulating that site. Although of smaller amplitude, these ADM MEPs showed specific modulations during the choice RT tasks. As such, we observed a significant suppression of ADM MEPs during the delay period. Interestingly, this suppression was systematically more pronounced when MEPs were elicited in a non-selected than in a selected hand, regardless of the task variant. In fact, the amount of ADM MEP suppression observed in a non-selected condition was similar to that observed in the (non-selected) FDI muscle. Hence, these results suggest that relevant and irrelevant muscles are suppressed to the same extent in a non-selected hand, which is consistent with early reports [16, 21] and with the view that this hand representation is targeted by a rather generic inhibitory influence [4, 5, 7]. In contrast, we observed a significant difference in the degree of MEP suppression for the ADM and FDI muscles in the selected condition, with MEPs being less suppressed in the ADM than in the FDI. This result suggests that the inhibitory influence directed at a selected representation (related to impulse control) is quite specific, reducing mostly the excitability of selected effectors and much less that of surrounding motor representations.

In conclusion, the present study demonstrates that MEP amplitudes elicited during the instructed-delay of choice RT paradigms are very sensitive to small variations to the task design. Based on our results, we think that the detection of motor inhibitory influences during action preparation requires the use of response devices that prevent subjects from responding prematurely (instead of standard keyboards) in order to reduce the influence of facilitatory process at the time motor inhibition is probed. Future studies are required to further identify how task features can impact on MEP suppression during action preparation.
